# Nurses experience of caring for patients with COVID-19: A phenomenological study

**DOI:** 10.3389/fpsyt.2022.922410

**Published:** 2022-07-19

**Authors:** Rasmieh Al-amer, Maram Darwish, Malakeh Malak, Amira Mohammed Ali, Kadejeh Al weldat, Abdulmajeed Alkhamees, Khaled S. Alshammari, Yacoub Abuzied, Sue Randall

**Affiliations:** ^1^Faculty of Nursing, Isra University, Amman, Jordan; ^2^School of Nursing and Midwifery, Western Sydney University, Penrith, NSW, Australia; ^3^Vascular Surgery Department, University Hospital of Wales, Cardiff, United Kingdom; ^4^Community Health Nursing, Faculty of Nursing, Al-Zaytoonah University of Jordan, Amman, Jordan; ^5^Department of Psychiatric Nursing and Mental Health, Faculty of Nursing, Alexandria University, Alexandria, Egypt; ^6^Department of Medicine, Unayzah College of Medicine and Medical Sciences, Qassim University, Unayzah, Al Qassim, Saudi Arabia; ^7^Stroke Unit, Nursing Department, Rehabilitation Hospital, King Fahad Medical City, Riyadh, Saudi Arabia; ^8^Spinal Cord Injury Unit, Nursing Department, Rehabilitation Hospital, King Fahad Medical City, Riyadh, Saudi Arabia; ^9^Susan Wakil School of Nursing and Midwifery, Faculty of Medicine and Health, The University of Sydney, Sydney, NSW, Australia

**Keywords:** nurses, experience, caring, COVID-19, collectivist, a phenomenological study, Arabic culture, Jordan

## Abstract

**Introduction:**

COVID-19 has impacted all dimensions of life and imposed serious threat on humankind.

**Background:**

In Jordan, understanding how nurses experienced providing care for patients with COVID-19 offers a framework of knowledge about similar situations within the context of Arabic culture.

**Aim:**

To explore nurses' experience with providing hands-on care to patients with active COVID-19 infection in an Arabic society.

**Methods:**

A descriptive phenomenological study interviewed 10 nurses through a purposive sampling approach until data saturation was reached. The research site was hospital designated to receive patients with active COVID-19 infection. Semi-structured interviews were used to collect the data.

**Findings:**

Three themes were generated from the data: the impact of the COVID-19 outbreak on nurses' health; unfamiliar work and social environments; and conforming to professional standards.

**Discussion:**

There are specific risks to the physical and mental wellbeing of nurses who provide hands-on care to patients with COVID-19 in an Arabic society.

**Implication for nursing and health policy:**

Health care institutions should consider establishing programs that promote nurses' wellbeing and support their productivity in a crisis. A danger pay allowance should be considered for nurses during extraordinary circumstances, such as pandemics.

## Introduction

The novel coronavirus-2 (SARS-CoV-2) virus emerged in Wuhan in late December 2019 and was declared as a pandemic by the World Health Organization (WHO) early in March 2020, and is the most serious virus outbreak yet in the 21st century (1). It has placed the global health care system and health workforce under unprecedented pressure. Health care systems worldwide have failed to provide effective preventive measures and management plans to contain the pandemic (2). This has promoted feelings of uncertainty among health care professionals, specifically nurses.

Nurses spend more time with patients than any other health care team; hence, it is plausible that they are at higher risk for attracting and transmitting the disease (3). The nature of the SARS-CoV-2 virus including its incubation period, mode of transmission, and infectivity emphasized the continuity of the threat and risk to nurses' health, justifying feelings of uncertainty and mental health concerns (4, 5).

COVID-19 outbreak has raised levels of stress, anxiety and depression among nurses. Recent studies from China (5–7) and Italy (8), the first two regions to be impacted by this disease, reported that nurses who delivered hands-on care for patients with COVID-19 were at higher risk of developing mental health disorders compared with other health care providers. Furthermore, a study by Rossi et al. (8), which included 1,379 health care providers, of which one-third were nurses, reported that having a co-worker who suffered from COVID-19 was a significant factor that exacerbated mental health problems among nurses.

## Background

In collectivist communities the dimensions of care are bound by the nature of the social norm, for example, family members who work as health care providers are obliged to provide care for their relatives who suffer an illness, even if there is a concurrent, potentially deadly, health threat like COVID-19. All aforementioned features of the SARS-CoV-2 virus and the nature of the Arabic cultural norms increase the susceptibility of nurses and other health care workers to the virus, thereby negatively impacting their mental health status (9, 10). For example, a collective social norm mandates that nurses who have a relative with COVID-19 should provide care for them and dedicate their time for the family, because a family's interest comes first.

Like other countries, Jordan had suffered from the COVID-19 pandemic, which has caused a wide range of physical, social, psychological and economic implications. Case zero in Jordan was reported on 2 March 2020 in the capital city of Amman, with the first COVID-19 carrier being a traveler from Italy (11). Thereafter, the incidence curve increased slightly and then plateaued, during which the Jordanian health authorities reported low incidence levels ranging from one case in the beginning of March 2020 to 68 cases in mid-August 2020. Afterwards, the curve started showing an upward trend, with the number of confirmed cases increasing significantly to 7,933, among whom 60 had died (1), including 29 health care providers (12). Although the Jordanian health authorities had a 7-month window (March–November) to enhance the health care system preparedness for this pandemic, progress remained slow. Two studies assessed nurses' reactions to COVID-19 in Jordan (9, 10). Al-Amer et al. (10) study recruited around 400 nurses using an online survey, and found a high prevalence of depression, anxiety and stress among the study participants. Abuhammad et al. (9) study identified nine Facebook nursing groups in Jordan and evaluated their accounts regarding their perception of their roles during COVID-19. Both studies were conducted between March and April 2020 when the number of cases ranged between 1 to 22 cases daily.

Few qualitative studies have investigated nurses' perception regarding the care for patients with COVID-19, particularly those with active symptoms. To our knowledge, no studies have considered how perceptions might differ for nurses living and working in a collectivist society such as the Jordanian's. Hence, understanding nurses' perception and experience in relation to the current pandemic situation carries substantial value. The present study is the first to explore the experiences of Jordanian nurses in the face of the COVID-19 pandemic. Hence, the study was guided by the following research question “What was the lived experience by Jordanian nurses caring for Patients with COVID-19 like?”

## Aim

In this research, we aimed to describe the experience of providing hands-on care to patients with COVID-19 from the accounts of nurses in Jordan which is a collectivist society. The term “active COVID-19” was conceptualized to refer to any person who tested positive for SARS-CoV-2 and exhibited symptoms that warranted hospital admission.

## Methods

### Study design

This study used the descriptive phenomenological approach by Husserl (13). According to Husserl, the phenomenon should be allowed to speak for itself through the voices of people. Husserl's (14) philosophy states that researchers must “bracket” any prior knowledge, preconceived notions, and judgements about the phenomenon of interest before data collection to avoid influencing outcomes (15). The researchers who collected and analyzed the data practiced phenomenological reduction by asking themselves a series of questions.

### Sample and setting

Using purposive sampling, 10 nurses who provided hands-on care for patients with active COVID-19 were approached. The purposive sampling technique was deemed suitable for this study because the data source was a closed fit to deliver data that would answer the study-related research question (16, 17). Nurses providing hands-on care for patients with COVID-19 are the most experienced and will be informed about this phenomenon. Thus, they were recruited to reflect the expert experience that can deliver data relevant to the research inquiries (18).

The study populations were all nurses and recruited from major hospitals in Amman that were designated for treating patients with COVID-19. The inclusion criteria for the study subjects were being a registered nurse providing hands-on care for patients with active COVID-19 infection at least a month prior to the data collection procedure, and willing to participate in this study. The study excluded associate nurses because their duty of care is limited to specific procedures, for example, they were not allowed to administer medications, and the study aimed to have participants that could reflect on the whole experience of caring for patients with COVID-19. The sample size was determined based on the saturation, which took place on the 10th participant when the collected data held no new additional findings (19).

### Data collection

The first author, who has a PhD in nursing and experience in conducting qualitative interviews collected the data using a semi-structured interview guide developed by the first and last author based on available literature (Box 1). The guide was piloted among two nurses and subsequently, slight modifications were made, bearing in mind that the two-pilot interviews were excluded from the final report because it aimed to improve interview schedules and specific questions. Literature have reported that pilot study in qualitative research could identify several challenges for researchers such as but not limited to the “instrumentation rigor” and management of bias (20, 21). Piloting the interview guide was an important step toward the decision that the data collection should be conducted using a telephone-based approach, rather than in person as was originally proposed. In the pilot, wearing face masks made establishing a rapport with the participants difficult because the masks concealed our identities. We tried to overcome this barrier by disclosing some information about our families because in collectivist communities, individuals would be honored and more accepting if their families have good reputations. Additionally, during the interviews, the participants displayed hospitality by providing food, which should not be refused; thus, we felt that this might harm us or harm our participants. Accordingly, we committed to uphold “non-maleficence,” as the wellbeing of the researchers, the community and the participants were a priority, and switched our approach to telephone-based interviews. It is important to note that COVID-19 has necessitated innovation in a wide range of dimensions of our lives—and research is no exception. For example, in qualitative research paradigm, face-to-face interviews were considered the “gold standard” approach to collecting data (17, 18, 22). However, during the COVID-19 pandemic, the face-to-face interview approach has encountered many constraints of social distancing and the prioritization of participants' and researchers' safety (23). Conducting qualitative interviews using a telephone approach offered researchers the opportunity to study the contexts of crisis while safeguarding participants and researchers (Roberts et al., 2021). However, telephone interviews could restrict the establishment of rapport between the researcher and the respondents which in turn could influence the richness of the data (22).

Box 1 **Participants interview guide**

- Can you tell me your experience of providing hands-on-care for a COVID-19 patient since the advent of the pandemic?
- Can you share with me an experience that of significance to you during providing hands-on-care for a COVID-19 patient?
- Please describe a day in which you were taking care of a COVID-19 patients.
- Please share me how do you see yourself as “a nurse” during this pandemic.

Ethics approval was granted by Isra University Human Research Ethics Committee/ethics approval number (JS/BA/94). Then an invitation was sent out to hospitals where patients with COVID-19 were treated. Hospitals that agreed to participate were asked to provide the research team with their nurse's email addresses, after which emails introducing the research team and the purpose of this study were sent to the nurses through hospitals “nursing departments” emails. Nurses who replied and fulfilled the inclusion criteria were contacted and sent a detailed participant information sheet and consent form. Signed consent was returned *via* email. The interviews, arranged at mutually convenient times, were semi-structured with pre-determined but open-ended questions that required rigor in the topics addressed but allowed flexibility in the exchange. The participants were advised that they could stop the interview at any point, and informed that the interview would be recorded using the “Voice Memos” mobile application for data collection and quality assurance purposes. Two nurses did not agree to their interview being recorded, prompting the first author to take down notes instead. Each interview lasted between 40 and 50 min.

The first author who is a female and shared the participants' language and culture conducted the interviews which facilitated establishing rapport with the participants. Participants were asked to narrate their experiences with providing hands-on care to patients with active COVID-19 (using their own words). Afterwards, each interview was transcribed verbatim. Another author reviewed and checked each transcribed interview against the audio materials. Identifying information was removed and participants were allocated a code. The reporting of data in this study has been performed according to the COREQ guidelines. All interviews took place between November and December 2020.

### Data analysis

Data collection and analysis occurred concurrently and provided “rich” quality rather than “thick” quantity of data (24). Three of the researchers convened after the analysis of each interview to validate the analytical process. Data analysis were performed manually and was guided by Colaizzi (25) framework for phenomenological data analysis, which included the following steps: (a) familiarization; the researcher read each interview transcript several times to familiarize themselves with the data; (b) identifying significant statements; all statements in the narrative that had direct relevance to providing care for patients with COVID-19 were identified; (c) Formulating meanings; meaning relevant to the studied phenomenon was extracted from a careful attention of important statements. (d) significant statements and identified meanings across all interviews were grouped into themes (e) exhaustive description was generated in which the researchers have written a complete and a comprehensive description of the phenomenon under study where all the themes were included; (f) the researchers then consolidated the exhaustive description into a short, and dense statement that weas important to the phenomenon under study and the basic structure was constructed; (g) the researcher validated the study findings by the participants. Afterwards, quotes were provided to support the themes.

### Rigor

This study maintained the trustworthiness of the data based on the criteria established by Lincoln and Guba (26), including credibility, transferability, dependability, and conformability. The credibility of data was ensured by “member checking” in which we have sent interviews transcripts to the participants for validation, however; only seven of them have responded. Also, the credibility was enhanced by the co-analyses that was jointly conducted by three of the research team. Also, this study provided a thick description of the context, the participants' characteristics, the settings, and the data collection procedure to maintain transferability. Dependability was ensured for the researcher kept a reflexive journal throughout the study and the data analysis process (27), Confirmability was confirmed by keeping a careful record” an audit trail” which includes the original notes, transcription and the analysis to preserve confirmability.

## Findings

### Participant characteristics

Ten nurses aged 27–45 years participated in the study (six were female), two contracted COVID-19 (one male and one female), nine had a bachelor's degree and one had a master's degree. Participants' experience as registered nurses ranged between 1 and 15 years.

Three major themes were obtained by the researchers through the analysis: The impact of the COVID-19 pandemic on nurses' health; Unfamiliar work and social environments; and conforming to professional standards.

#### Theme 1: The impact of the COVID-19 pandemic on nurses' health: “I am at a huge risk of getting infected and ending up in the ICU and dying”

Nurses strongly perceived COVID-19 as a deadly disease that negatively impacted their physical and psychological health. Moreover, they were uncertain regarding how this virus would behave. They felt they were at greater risk of infection,

“*I did not recognize my friend when he got it, I am too afraid, no strong treatment for it [G]*

Three of the participants felt that COVID-19 was a serious physical threat, not only to them, but also to others around them, given that they might become potential agents of transmission:

“*I moved my daughter to my mother-in-law's place because my wife and I are both working. There will be a chance she will end up being a COVID-19 patient because of us.” [H]*

The threat posed by this virus included physical implications and psychological difficulties. Eight of the participants felt that the health of their patients did not improve and instead deteriorated. They felt depressed about the situation and uncertain about their own health:

“*I feel helpless and depressed. No matter what I do, the results are not encouraging; people dying every day, few of them made it.” [M]*

When health care workers became infected and exhibited active symptoms that required intensive care and ventilator support, participants expressed having experienced some form of physical pain, somatisation and helplessness. Six of the study participants remarked that seeing a colleague infected with COVID-19, being on ventilator was most depressing.

“*It was depressing to see one of the anaesthesiologists connected to the ventilator, I (felt) a crushing sensation on my chest. I could not do anything.” [A]*

Most participants remarked that they were stressed, anxious and scared from the uncertainty and often used the phrase “what if,” alongside their fear of transmitting it to their families.

“*I keep on thinking, what if I got infected or transmitted the disease to my family; this causes me anxiety and stress; I can't run from such thoughts.” [R]*

Having a family member who got infected brought up severe psychological reactions that were compounded by guilt. The nurses involved assumed that they were the source of this infection and felt extremely depressed:

“*When my father got infected with COVID-19, I was badly depressed I kept on blaming myself. What if my father dies?” [M]*

Being asymptomatic would not help health care providers escape blame, shame and stigma. However, the participants admitted that these were the features of this pandemic:

“I personally got infected with COVID-19 on 21 October 2020. Three days before I knew I had corona, I visited my uncle. On 24 October, he had symptoms, and he has been on oxygen therapy. He kept on calling me and telling me that I have infected him, as if I am to be blamed. I did not know I had corona when I visited him, and the whole family were stigmatizing me as a person who transmitted it to others.” [T]

#### Theme 2: Unfamiliar work and social environments: “Everything is changing in all dimensions; the workplace, and socially”

Nurses felt that COVID-19 had altered the work and social environment. COVID-19 was viewed as a disease that had implications on social relationships and interpersonal communication through their social life in general. All these changes were new to collectivist communities in which gatherings are main pillars of social life. Furthermore, nurses' workplaces experienced dramatic challenges that included the use of unfamiliar and limiting personal protective equipment (PPE) as the only protective measure against contracting the virus. Moreover, nurses had to address a wide range of new medical procedures and drugs.

Participants stated that they lacked PPE as well as the knowledge needed to provide quality care. No clear strategy or guideline had been established. Continuous changes in the treatment plan resulted in inefficient care:

“*The lack of PPEs, the care we were providing were not anchored on a solid guideline. They changed the protocol many times, this will reduce the quality of care.” [M]*

They reported having to address new PPE measures, which had been portrayed as a physical and psychological burden on nurses; donning and doffing were a daily struggle.

“*I personally put my PPEs on and change these PPE twice a day, I postpone everything that I need to do to a PPE-free slot once a day. It is very exhausting to keep donning and doffing.” [G]*

Four participants remarked that whilst wearing PPE during fasting for Ramadan, they experienced severe unbearable headaches, mainly due to dehydration:

“*During Ramadan, I was fasting and had to wear the PPEs, and I felt thirsty because it is a hot outfit…after that I developed a headache, which affected my capacity to provide care.” [N]*

Masks and face shields were perceived as barriers to communication. Participants mentioned that patients often could not hear them clearly and would ask them to repeat their words multiple times:

“*I got frustrated to repeat what I have just said, it was very difficult for me to connect with my patients, how a nurse would be able to build a relationship with patients.” [Z]*

All participants reported that patients felt frightened by nurses wearing PPE. It created a barrier that exacerbated loneliness, which could have resulted in severe anxiety and worries for patients and nurses. In addition, patients had difficulty in accepting personnel in PPE.

“*My patients reported feeling lonely, they were not familiar with seeing nurses behind shields.” [S]*

Participants experienced unfamiliar social environments and reported that they encountered limitations when visiting their parents and elderly family members. The pandemic prevented them from paying the utmost cultural respect to their parents. Participants said keeping distance from their parents or any elderly family members during normal conditions brought shame. However, they practiced social distancing and limited their visits to their parents, given that they were obliged by COVID-19 restrictions and wanted their parents to survive:

“*It is hard to feel that you are trying to keep some social distancing with your parents. It is important to greet them with respect, hug them, kiss their hands and show them great admiration.” [D]*

#### Theme 3: Conforming to professional standards: “This is my career, I should serve no matter what, and I have to care.”

Although nurses were aware of the risk imposed by this pandemic and most of them remarked that they have suffered from physical and psychological stress, they were all committed to nursing ethics and continued to care for infected patients. They also stated that caring for infected patients was formed partly because of their religion, as well as the nature of their career.

“*I am a Muslim; I do everything for God; I wanted my patients to get better so I can take care of the others who are on the waiting list.” [H]*

However, all participants reported a time when they felt ambivalent toward providing care for infected patients, particularly when a shortage of PPE occurred, and some of the staff had contracted the virus. However, they continued to provide care and fight against COVID-19. They stated that they provided care and found a refuge in God for protection because of their good deeds:

“*I had thoughts ‘I am not a martyr,’ but I felt this is not me, I am a person ‘who accepted to be identified as a nurse,’ so, I decided to act as a nurse in accordance with my career ethics.” [Z]*

Some of the participants stated that caring for patients with COVID-19 had created an ethical dilemma: personal safety vs patient wellbeing. On the personal side, nurses were reluctant to provide care when PPE were lacking, and their safety was jeopardized. They argued that they had an ethical commitment toward their own safety and denied the widespread perception that nurses were born to be martyrs:

“*I had a huge conflict and stress in me, but when they made the protective gears available for us, we did what our conscience required us to do, we cared.” [N]*

## Discussion

The current study aimed to explore how Jordanian nurses experienced providing care to patients with active COVID-19. In Jordan, work and family commitments are intertwined. Three themes were identified: the impact of COVID-19 on nurses' health; unfamiliar work and social environments; and conforming to professional standards.

COVID-19 has placed the nursing workforce across numerous countries under unprecedented pressure, which in turn has impacted their mental wellbeing (3). The current study supports previous literature which reported that nurses had a high level of depression, anxiety and stress during the COVID-19 pandemic (10) and viewed COVID-19 as a deadly disease (4–7). This could lead nurses to perceive COVID-19 as a disease with high fatality rate, hence, thinking of caring for infected patients as a burden. Nurses who provide care for patients of Arabic culture are more prone to mental health difficulties because such societies spend large amounts of time with their extended families and are not familiar with social distancing, specifically when it comes to their relatives (10).

The findings in the current study highlight the negative impact on participants' physical and psychological wellbeing. Some study participants experienced vicarious trauma after witnessing health care colleagues' sufferings and/or death from SARS-Cov-2 infection. This kind of trauma could be viewed as the “cost of caring” as nurses witness their colleagues suffering, this has resulted in psychological, and physiological difficulties. It appears that Jordanian nurses have become occupied with thoughts about their associates with COVID-19 infection. Similar findings were reported among Italian health care providers (8). Literature describes this as secondary traumatic stress syndrome (7). One participant described the threat to health in terms of actual physical pain of seeing colleagues being ventilated. We argue that nurses can live vicariously with the pandemic even when not infected with COVID-19. For example, repeated exposure to traumatic experiences could lead to vicarious trauma (28, 29), emotional distress and compassion fatigue (30, 31), which was also noted in nurses caring for COVID-19 patients (32). The relentless nature of the pandemic adds to this risk and was recognized by participants as being compounded by unfamiliar environments. These findings suggest that nursing leaders and policy makers need to pay more attention to the psychological capacity of nurses going forward.

Acute concern among nurses regarding the safety of their family members and the potential of transmitting the disease to their families was evident from the current study; this is understandable in light of the fact that nurses are responsible to promote their own health safety, and in Arabic community family's interest takes precedence on individual's benefits. Hence, nurses are required to balance their obligations of beneficence and duty to care for patients and duties to protect their loved ones. Prioritizing the wellbeing of family has been reported as a barrier to health care workers' motivation to work in a pandemic (33). Although this was not evident in the present study, the effects of working during a pandemic emphasized the psychological distress experienced by our participants. This was further highlighted when one participant was blamed for the illness of a relative who contracted COVID-19. Several editorials have identified factors that would increase the propensity for a health care worker to develop mental health disorders during this outbreak, including fear of catching COVID-19 or transmitting it to a loved one (30, 34) and the high rates of associated mortality (34). Our study supports these commentaries. Our participants reported experiencing negative emotions when they witnessed the long-term suffering of their family members because of COVID-19.

Stresses at work can be mitigated by a healthy work–life balance. Having interests outside work, being able to socialize (35), and family and social support are important factors in reducing mental illness and burnout (36). In Arabic societies, the family is viewed as part of oneself. Thus, the pandemic negatively affected social norms on family inclusion. For example, in Arabic societies such as Jordan, the importance of family cannot be overstated. Large family gatherings and the social etiquette of hugging and kissing, especially with elderly relatives, has been considered a sign of utmost respect. Removing these facets of life that serve as a buffer against a challenging work environment as a result of pandemic measures (e.g., social distancing and lockdown), have added an additional layer of risk for Jordanian nurses to develop current and future mental health problems.

Wearing PPE was viewed as a barrier between the nurse and patient and negatively impacted communication with both patients and colleagues. This was plausible because verbal and non-verbal communication are both equally important in establishing rapport and trust. In line with our findings, McCarthy et al. (37) reported that wearing PPE hampered effective communication with patients, specifically among patients who were in isolation (37, 38). For example, the face mask has made non-verbal and verbal communication difficult. Face masks were also viewed as a physical barrier to empathy, which is essential in developing trust, a therapeutic relationship and effective communication between health care providers and patients because masks concealed their identity and facial expressions (39). Overall, PPE was found to conceal the role of a health care provider. For example, some patients had difficulty distinguishing a nurse from other health care providers, adding another barrier to effective communication and impacting patient–nurse relationships (37). However, we believe PPE are very important for patients and the nurses' health; in addition, employers are required to provide nurses with adequate PPE, and the institutions should be held accountable for any harm that affects nurses due to the lack PEE.

Nursing during COVID-19 has been recognized as a challenge to nurses' wellbeing (40). Our participants described changes in their work environments that caused additional work given that they were inexperienced and lacked education regarding the changes and how to appropriately manage them. In ordinary circumstances, strong leadership is expected to steer staff through change. A report by The King's Fund (41) stated that nurses and midwives have three core work needs: (1) autonomy (control over their work lives and ability to act consistently with their values); (2) belonging (the need to be connected to, cared for and be caring of others at work and to be respected, valued and supported); and (3) contribution (the need to be effective in their work and to deliver outcomes that are valued). Further recommendations suggest that commitment across health regulators, health improvement bodies and all partners in health and social care are required, that, alongside the core work needs, would ensure wellbeing and motivation at work and minimize stress (41). A framework such as this may be a valuable tool for supporting staff in future work environments as they recover from the COVID-19 pandemic and prepare for future challenges in the health sector.

Belonging (1) and contribution (3) (41) were evident in our participants responses under the theme “conforming to professional standards,” Commitment to their professional and moral responsibilities as a nurse were clearly articulated. This may be explained in terms of the courage of compassion (41). A strong religious belief regarding what was right in this situation was also evident. However, despite their commitment and religious beliefs, participants did struggle with the ethical dilemma of balancing their responsibility to themselves and their family with the people in their care. The dilemma was exacerbated by the magnitude and uncertainty of the pandemic. Work overload, which was herein caused by COVID-19, has been reported as one reason for the occurrence of ethical dilemmas in nursing practice (42).

## Conclusion

In exploring nurses' experiences of caring for COVID patients, we discovered that nurses working and living in a collectivist society, in which the work and social life are highly interconnected reported high levels of mental distress. Although nurses had suffered from physical, social, and mental difficulties, they were committed to providing patient care in which they conformed to their professional standards. The balancing act of managing personal wellbeing, societal expectations and professional commitments added to the impact of caring for COVID-19 patients in a collectivist society in which resources and health management structures to keep nurses safe were limited. The findings have implications for nursing and health policy.

## Implications for nursing and health policy

Findings presented herein are significant in the context of being prepared for a situational crisis. This study has identified that nurses experienced risks to mental health because of COVID-19, hence, nursing leaders should develop ways to protect their staff from stress created in such situations. Health care institutions should consider establishing counseling programmes that promote nurses' mental health and support their productivity in a crisis, with specific emphasis on self-care activities The risk to health perceived by participants suggests that policymakers should have a plan in place to guide nurses in the present and future outbreaks. For instance, update nurses with all the current guidelines that are issued by health care bodies such as the (WHO) and provide them with all protective supplies to stay informed, productive and connected. Based on the findings from this study, nurses could be consulted for more practical PPE designs to facilitate their movements and establishing a connection with their patients.

Nurse leaders could use these findings to increase the awareness of the danger that nurses may encounter during such crises and request “a danger pay allowance” to provide them with additional compensation during exceptional times.

### Study limitation

This study focuses on Arab nurses from collectivist communities. Other nurses with different ethnicities may have different experiences with providing care to patients with COVID-19. Furthermore, the study used the telephone approach interview, although this approach has a wide range of advantages such as but not limited to overcoming the geographic limitations, it could have limited cultivating the rapport and connection between the interviewer and the interviewee because of the absence of visual or non-verbal cues which in turn could have affected the richness of the interviews.

## Data availability statement

The raw data supporting the conclusions of this article will be made available by the authors, without undue reservation.

## Ethics statement

The studies involving human participants were reviewed and approved by Isra University Ethics Committee (no. JS/BA/94). The patients/participants provided their written informed consent to participate in this study.

## Author contributions

All authors listed have made a substantial, direct, and intellectual contribution to the work and approved it for publication.

## Conflict of interest

The authors declare that the research was conducted in the absence of any commercial or financial relationships that could be construed as a potential conflict of interest.

## Publisher's note

All claims expressed in this article are solely those of the authors and do not necessarily represent those of their affiliated organizations, or those of the publisher, the editors and the reviewers. Any product that may be evaluated in this article, or claim that may be made by its manufacturer, is not guaranteed or endorsed by the publisher.
